# Do we need to hold aspirin before vitreoretinal surgery? A systematic review and meta-analysis

**DOI:** 10.1186/s40942-026-00840-3

**Published:** 2026-03-31

**Authors:** Hashem Abu Serhan, Abdullah Ahmed, Muhammad M. Elsharkawy, Ameen Alkhateeb, Mutaz Al-Nawaflh, Husam Abu Dawood, Anant Pai

**Affiliations:** 1 Department of Ophthalmology, Hamad Medical Corporation, Doha, Qatar; 2 Department of Ophthalmology and Visual Sciences, University of Illinois Chicago, Chicago, IL USA; 3 Faculty of Medicine, Alexandria University, Alexandria, Egypt; 4 Department of Ophthalmology, King Hussein Medical Center, Amman, Jordan; 5 Faculty of Medicine, Palestine Polytechnic University, Wadi Al-Hariyyah, PO: P720, Hebron, Palestine

**Keywords:** Aspirin, Antiplatelet therapy, Vitreoretinal surgery, Vitrectomy, Hemorrhagic complications

## Abstract

**Purpose:**

To evaluate the safety of aspirin continuation versus discontinuation in patients undergoing vitreoretinal surgery by assessing hemorrhagic complications and visual outcomes.

**Methods:**

The review protocol was prospectively registered in PROSPERO (CRD420251270694). A systematic review and meta-analysis was conducted following PRISMA guidelines. PubMed, Scopus, Ovid, and Web of Science were searched from inception through August 31, 2025. Studies comparing aspirin continuation versus discontinuation or no aspirin use in adult patients undergoing vitreoretinal surgery were included. The primary outcome was vitreous hemorrhage. Secondary outcomes included hyphema, subconjunctival hemorrhage, subretinal hemorrhage, choroidal hemorrhage, retinal hemorrhage, and visual acuity changes. Risk ratios (RR) with 95% confidence intervals (CI) were calculated using random-effects models. Quality assessment was performed using the Newcastle-Ottawa Scale, and certainty of evidence was rated using the GRADE framework.

**Results:**

Seven studies comprising 2,889 surgical procedures (2,873 patients) were included. Aspirin continuation was not associated with significantly increased risk of vitreous hemorrhage (RR 1.55, 95% CI: 0.73–3.29, *p* = 0.253, I²=61.7%), hyphema (RR 1.52, 95% CI: 0.56–4.17, *p* = 0.415, I²=0.0%), subconjunctival hemorrhage (RR 1.20, 95% CI: 0.58–2.48, *p* = 0.622, I²=0.0%), subretinal hemorrhage (RR 1.06, 95% CI: 0.15–7.63, *p* = 0.956, I²=82.4%), choroidal hemorrhage (RR 2.07, 95% CI: 0.81–5.24, *p* = 0.127, I²=0.0%), retinal hemorrhage (RR 1.68, 95% CI: 0.33–8.52, *p* = 0.532, I²=73.6%), or postoperative visual acuity (MD = 0.26 logMAR, 95% CI: −0.005 to 0.52; *p* = 0.055). Included studies were rated as moderate to high quality on the Newcastle-Ottawa Scale. GRADE certainty of evidence was rated as low to very low across all outcomes.

**Conclusions:**

Aspirin continuation appears safe in vitreoretinal surgery, with low risk of hemorrhagic complications. Given aspirin’s thrombotic benefits, it can likely be continued in most patients. However, this conclusion, based on low-certainty evidence, should be interpreted with caution and requires confirmation from high-quality prospective randomized trials.

**Supplementary Information:**

The online version contains supplementary material available at 10.1186/s40942-026-00840-3.

## Introduction

Vitreoretinal surgery encompasses a broad spectrum of procedures for treating retinal detachments, diabetic complications, epiretinal membranes, and macular holes. The volume of these procedures has grown substantially, with pars plana vitrectomy increasing more than fourfold over the past two decades in developed countries [1]. Rhegmatogenous retinal detachment alone affects approximately 10 to 18 per 100,000 individuals annually in the United States [2], representing just one of many conditions requiring vitreoretinal intervention. As the global population ages and surgical techniques continue to advance, the demand for these vision-saving procedures continues to rise.

Patients undergoing vitreoretinal surgery frequently receive antiplatelet therapy for cardiovascular disease prevention. Aspirin remains one of the most widely prescribed medications globally, with approximately one-third of U.S. adults aged 40 years and older currently taking it for cardiovascular protection [3]. Notably, nearly half of adults aged 70 years and older — a population that frequently requires vitreoretinal surgery — report using aspirin for primary prevention alone [4]. This high prevalence of antiplatelet therapy creates a common clinical dilemma when these patients require surgical intervention.

The perioperative management of aspirin therapy presents a challenging balance between competing risks. Discontinuing aspirin exposes patients to potentially serious thromboembolic complications, including myocardial infarction, stroke, and stent thrombosis, with median onset times as brief as 8.5 days following cessation [5]. Some evidence suggests a “rebound” phenomenon following aspirin withdrawal, characterized by increased platelet reactivity that creates a prothrombotic state [6]. Conversely, continuing aspirin during surgery raises concerns about increased bleeding risk, which could be particularly consequential in the confined intraocular space where hemorrhagic complications might threaten vision.

Current evidence regarding aspirin management in vitreoretinal surgery remains conflicting and inconclusive. Several studies have suggested that aspirin continuation does not significantly increase the risk of severe hemorrhagic complications [7, 8], while others have reported higher rates of potentially sight-threatening bleeding events in patients receiving antiplatelet therapy [9]. The available literature is limited by small sample sizes, heterogeneous patient populations, varied surgical indications, and inconsistent reporting of whether antiplatelet medications were actually discontinued perioperatively [10]. Unlike cataract surgery, where recent large-scale meta-analyses have helped clarify aspirin management [11], no comprehensive synthesis of evidence exists specifically for vitreoretinal procedures.

This systematic review and meta-analysis aims to address this critical knowledge gap by evaluating the safety of aspirin continuation versus discontinuation in patients undergoing vitreoretinal surgery, focusing on both hemorrhagic complications and visual outcomes.

## Methods

### Protocol and registration

This systematic review and meta-analysis study was conducted in accordance with the methodological guidelines outlined in the Cochrane Handbook for Systematic Reviews of Interventions [12] and followed the reporting standards of the Preferred Reporting Items for Systematic Reviews and Meta-Analyses (PRISMA) Statement [13]. The review protocol was prospectively registered in PROSPERO (registration number: CRD420251270694). This review adhered to the tenets of the Declaration of Helsinki, and an institutional review board exemption was received for this study, given the use of previously published data.

### Search strategy

A comprehensive systematic search was conducted by two independent reviewers [AA and MA] across PubMed (MEDLINE), Ovid (MEDLINE and Embase), Scopus, and Web of Science from database inception through August 31, 2025, without language restrictions. The search strategy combined Medical Subject Headings (MeSH) terms and free-text keywords capturing vitreoretinal surgical procedures (vitrectomy, pars plana vitrectomy, retinal detachment surgery, macular hole surgery, epiretinal membrane surgery, scleral buckling), antiplatelet agents (aspirin, acetylsalicylic acid, platelet aggregation inhibitors), and perioperative management and hemorrhagic complications. Complete search strategies are provided in Supplementary Table S1.

### Inclusion and exclusion criteria

This review was structured according to the PICOS framework: Population (P) — adult patients (≥ 18 years) undergoing vitreoretinal surgery; Intervention (I) — perioperative continuation of aspirin therapy; Comparison (C) — aspirin discontinuation or no antiplatelet therapy; Outcomes (O) — hemorrhagic complications (vitreous hemorrhage, hyphema, subconjunctival hemorrhage, subretinal hemorrhage, choroidal hemorrhage, retinal hemorrhage) and visual acuity changes; Study design (S) — randomized controlled trials, prospective cohort studies, and retrospective cohort studies.

For this review, “continuation” was defined as uninterrupted aspirin use through the perioperative period with no modification to the treatment regimen, while “discontinuation” was defined as cessation of aspirin at least 3–7 days prior to surgery as operationalized in the original studies. Aspirin doses across included studies ranged from 80 to 100 mg/day. No restriction was placed on whether aspirin was used for primary prevention (e.g., prophylactic use in the absence of established cardiovascular disease) or secondary prevention (e.g., following myocardial infarction, stroke, or coronary stent placement); both indications were eligible for inclusion.

Studies were included if they met all criteria: original research investigating adult patients (≥ 18 years) undergoing vitreoretinal surgery (pars plana vitrectomy, scleral buckling, or combined procedures); comparison of aspirin continuation versus discontinuation, or aspirin use versus no antiplatelet therapy perioperatively; reporting of hemorrhagic complications (vitreous hemorrhage, hyphema, subconjunctival hemorrhage, subretinal hemorrhage, choroidal hemorrhage, retinal hemorrhage) or visual acuity outcomes; randomized controlled trials, prospective cohort studies, or retrospective cohort studies; sufficient data to calculate effect estimates; and English language publication.

Studies were excluded if they met any criterion: case reports or case series with < 20 patients; studies examining only other antiplatelet agents or anticoagulants without aspirin-specific data; exclusively pediatric populations; only cataract or anterior segment procedures; no comparison group; conference abstracts without full-text publication; review articles, editorials, or letters; unclear aspirin continuation/discontinuation status; or duplicate publications. When studies reported both aspirin and other agents, aspirin-specific data were extracted when available.

### Study selection process

Records identified through database searches were imported into EndNote X9, and duplicates were removed. Two independent reviewers [AA and MA] screened titles and abstracts using predefined eligibility criteria in Rayyan systematic review software [14]. Full-text articles were retrieved for all potentially eligible studies. Two reviewers [AA and MA] independently assessed full-text articles using a standardized eligibility form. Disagreements were resolved through discussion or consultation with a third senior reviewer [HAS]. The study selection process is documented following PRISMA guidelines.

### Data extraction

Data extraction was performed independently by two reviewers [AA and MA] using a standardized, piloted form. Extracted data included study characteristics (author, year, country, design, setting, sample size), population characteristics (age, gender, surgical indications, comorbidities), intervention details (aspirin dosage, timing of discontinuation/resumption), surgical details (procedure type, vitrectomy gauge, anesthesia type), and outcomes (hemorrhagic complications, visual acuity). Discrepancies were resolved through discussion or third-reviewer consultation [HAS]. Authors were contacted when data clarification was needed.

### Quality assessment and risk of bias

Methodological quality of observational studies was independently assessed by two reviewers [AA and MA] using the Newcastle-Ottawa Scale (NOS), evaluating selection of study groups (maximum 4 stars), comparability (maximum 2 stars), and outcome assessment (maximum 3 stars). Studies were categorized as high quality (7–9 stars), moderate quality (4–6 stars), or low quality (0–3 stars). For randomized controlled trials, the Cochrane Risk of Bias 2.0 tool was used. Disagreements were resolved through discussion or third-reviewer consultation [HAS].

### Outcomes

The primary outcome was vitreous hemorrhage risk, defined as postoperative bleeding into the vitreous cavity sufficient to obscure fundus details or require intervention. Given that included studies used heterogeneous definitions of vitreous hemorrhage — ranging from any clinically visible blood to hemorrhage specifically requiring surgical reintervention — each study’s own outcome definition was applied, and this heterogeneity in definition is acknowledged as a potential source of variability across studies. Secondary outcomes included hyphema, subconjunctival hemorrhage, subretinal hemorrhage, choroidal hemorrhage, retinal hemorrhage, and visual acuity changes (mean final visual acuity in logMAR at longest follow-up). All hemorrhagic complications were analyzed as binary outcomes (present/absent) using study authors’ definitions and grading systems. Subconjunctival, subretinal, choroidal, and retinal hemorrhage were analyzed as a combined outcome with subgroup analyses by hemorrhage type, mirroring the approach used for vitreous hemorrhage.

### Statistical analysis

All analyses were performed using R software version 4.4.3 with “meta,” “metafor,” “dplyr,” and “ggplot2” packages. For visual acuity outcomes, data reported in Snellen fractions were converted to logMAR equivalents using the standard formula (logMAR = − log10[Snellen fraction]). Non-Snellen values such as counting fingers (CF), hand movements (HM), and perception of light (PL) were converted using conventional logMAR equivalents of 1.85, 2.30, and 2.70, respectively, when reported. Risk ratios (RRs) with 95% confidence intervals (CIs) were calculated for binary outcomes; mean differences (MDs) with 95% CIs for continuous outcomes. Random-effects models using the DerSimonian-Laird method were employed, with Mantel-Haenszel pooling for binary outcomes and inverse variance for continuous outcomes. Heterogeneity was assessed using Cochran’s Q and I² statistics, interpreted per Cochrane guidelines. Pre-specified subgroup analyses for vitreous hemorrhage examined comparison strategy, study design, geographic region, and surgical indication. For the combined hemorrhagic complication outcome, subgroup analyses were performed by hemorrhage type (subconjunctival, subretinal, choroidal, and retinal hemorrhage). Sensitivity analyses using leave-one-out methods were performed for outcomes with high heterogeneity (I² > 50%). Publication bias was assessed using Doi plots and the Luis Furuya-Kanamori (LFK) index for all outcomes. Statistical significance was set at α = 0.05 (two-tailed). When studies reported zero events in one or both groups, a continuity correction of 0.5 was applied. Absolute risk differences (ARDs) with corresponding numbers needed to harm (NNH) were calculated for all binary outcomes using the control group event rate as the baseline, to contextualize the clinical relevance of the observed effect estimates. The certainty of evidence for each outcome was formally assessed using the GRADE (Grading of Recommendations, Assessment, Development and Evaluations) framework.

## Results

**Fig. 1:**
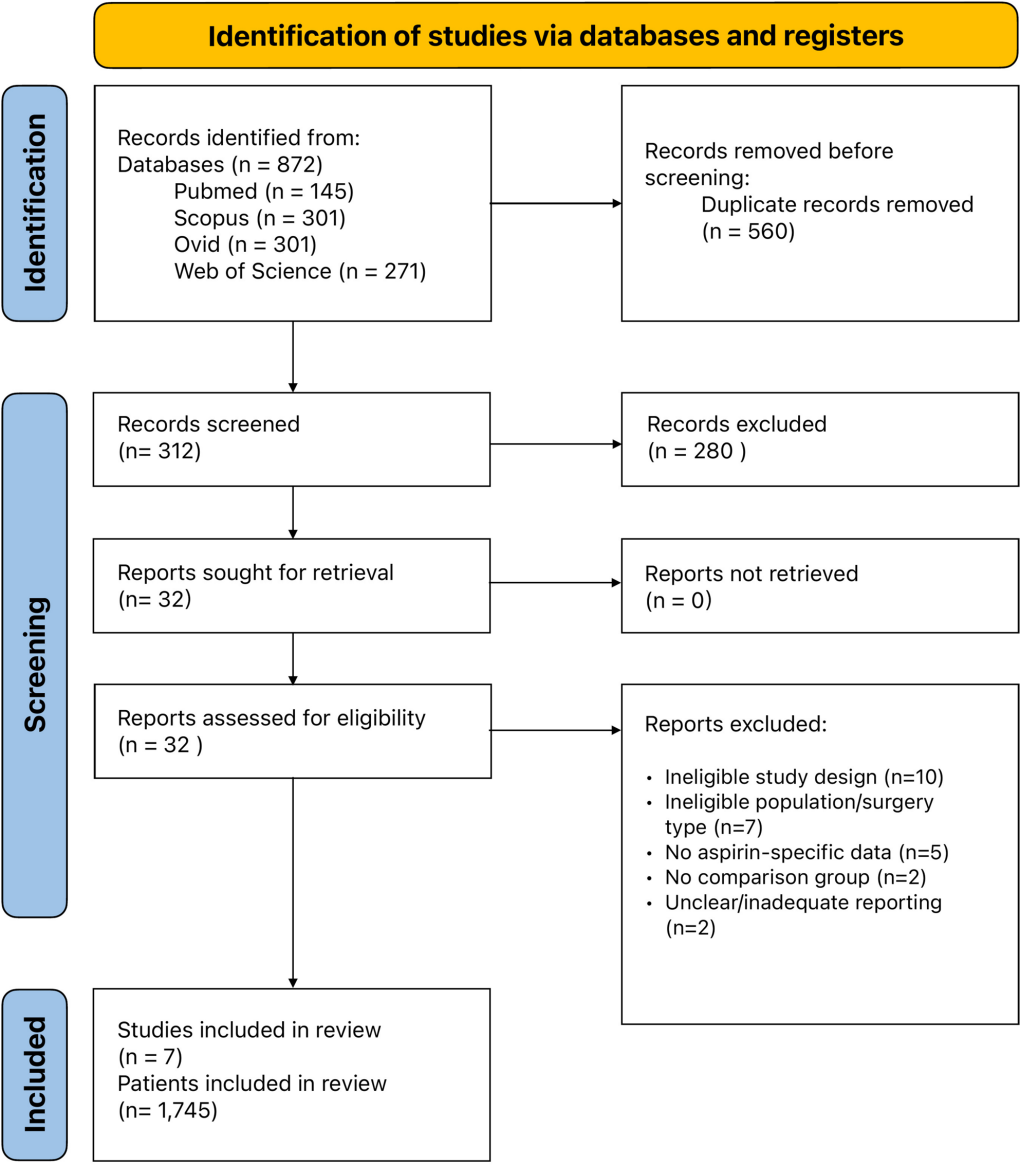
The PRISMA flowchart of included studies

The systematic literature search identified 872 records across four databases: PubMed (*n* = 145), Scopus (*n* = 301), Ovid (*n* = 155), and Web of Science (*n* = 271). After removing 560 duplicates, 312 records were screened by title and abstract, with 280 excluded. Thirty-two full-text articles were assessed for eligibility. Twenty-five reports were excluded due to ineligible study design (*n* = 10), ineligible population or surgery type (*n* = 7), no aspirin-specific data (*n* = 4), no comparison group (*n* = 2), and unclear or inadequate reporting (*n* = 2). Ultimately, seven studies met the inclusion criteria and were included in the meta-analysis (Fig. 1).

### Baseline characteristics of included studies

A total of seven studies comprising 2,889 surgical procedures (2,873 patients) were included in this systematic review and meta-analysis (Table 1). The included studies were published between 2003 and 2020, representing nearly two decades of evolving surgical techniques and perioperative management strategies. Study designs varied, with five prospective studies [15–18], one retrospective cohort study [19], and one retrospective case series [20]. The studies originated from diverse geographical locations, including France (two studies [16, 17]), China [18], the United Kingdom [7], Germany [19], Iran [15], and Australia [20], reflecting international practice patterns.

**Table 1:** Characteristics of included studies

Study	Year	Country	Design	Patients (*n*)	Aspirin/AP group (*n*)	Control/discontinuation (*n*)	Mean age (years)	Male (%)	Surgical indication	Surgical technique	Anesthesia	Follow-up	Primary outcome
Narendran [7]	2003	UK	Prospective cohort	541	60 (aspirin)	481	56.9	60.8	Mixed vitreoretinal	Various PPV/buckling	Mixed	Variable	Hemorrhagic complications
Fabinyi [20]	2011	Australia	Retrospective case series	155 eyes (139 patients)	68	87	63.0	64.7	Diabetic eye disease	PPV	Retrobulbar/general	1–3 months	Persistent VCH
Passemard [17]	2012	France	Prospective case series	206	44 (AP), 12 (AC)	144	67.5 ± 13.7	39.6	Mixed vitreoretinal	PPV (87.4%)	Peribulbar	1–10 months	Hemorrhagic complications
Ajudani [15]	2017	Iran	Prospective cohort	180	90 (aspirin)	90	60.5 ± 9.9	45.6	Proliferative diabetic retinopathy	20-gauge PPV	Retrobulbar	1 month	Bleeding complications
Meillon [16]	2018	France	Prospective multicenter	804	148 (AP), 63 (AC), 7 (both)	586	66.0 ± 9.5	56.3	Mixed vitreoretinal	PPV (87.4%), 23-G (21.9%), 25-G (71.9%)	Peribulbar	1 month	Hemorrhagic complications
Bemme [19]	2020	Germany	Retrospective single-center	893	140 (aspirin)	701	61.0 ± 14.0	60.4	Rhegmatogenous retinal detachment	PPV, buckling, combined	Mixed	Perioperative	Perioperative hemorrhages
Wang [18]	2020	China	Prospective randomized	110	33 (continuation), 34 (discontinuation)	43	65.7 ± 9.0	54.5	Mixed vitreoretinal	23-gauge PPV	Retrobulbar	3 months	Hemorrhagic complications

Abbreviations: AP, antiplatelet agents; AC, anticoagulant agents; PPV, pars plana vitrectomy; VCH, vitreous cavity hemorrhage; UK, United Kingdom; USA, United States of America

Note: Some studies included both antiplatelet and anticoagulant groups; data presented reflects aspirin/antiplatelet groups when available. Age presented as mean ± standard deviation when reported. Control groups include both aspirin discontinuation and no aspirin use depending on study design

Sample sizes ranged considerably from 110 patients in the smallest prospective randomized trial [18] to 893 patients in the largest retrospective cohort [19]. The comparison strategies varied across studies: some compared aspirin or antiplatelet use versus no antiplatelet therapy, while others compared aspirin continuation versus discontinuation. Several studies included patients on both antiplatelet and anticoagulant agents, with Passemard et al. [17] reporting 6 patients and Meillon et al. [16] reporting 7 patients on combined therapy. These patients on dual therapy were analyzed separately in the original studies but were generally excluded from the primary comparison groups in this meta-analysis. The mean age across studies ranged from 60.5 to 67.5 years, with male patients comprising 42.9% to 60.4% of study populations.

Surgical indications were heterogeneous and included rhegmatogenous retinal detachment, proliferative diabetic retinopathy, epiretinal membrane, macular hole, and vitreous hemorrhage from various etiologies. Surgical techniques also varied, with most studies employing standard pars plana vitrectomy using 20-gauge or 23-gauge instrumentation, while some included scleral buckling procedures with or without subretinal fluid drainage. Anesthetic approaches differed across studies, with retrobulbar, peribulbar, and general anesthesia all represented. The prevalence of systemic comorbidities varied, with diabetes mellitus reported in 15.9% to 54.5% of patients and hypertension in 34.8% to 75.8% across studies. Follow-up duration ranged from one week to several months postoperatively, with most studies reporting outcomes at one month. Notably, the control groups were defined differently across studies: some studies used patients not on any antiplatelet or anticoagulant therapy as controls [7, 15, 19], while others compared aspirin continuation versus discontinuation [18, 20], creating two separate comparison frameworks that were analyzed as subgroups in the vitreous hemorrhage outcome.

### The risk of vitreous hemorrhage post-vitreoretinal surgery

**Fig. 2:**
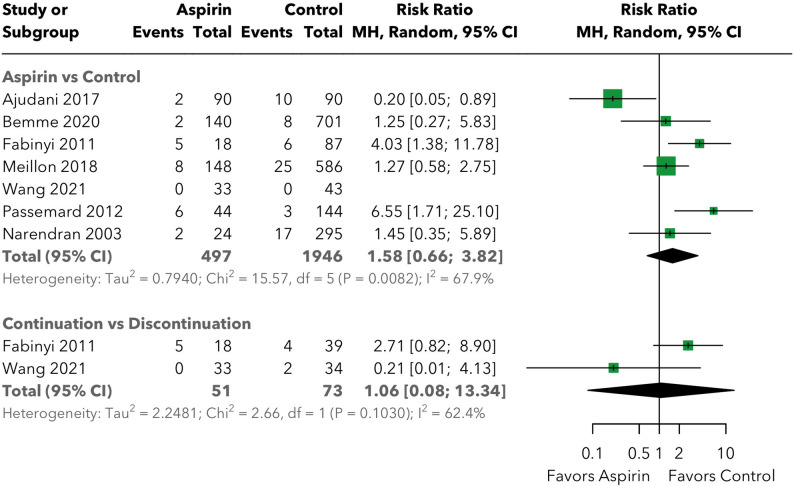
The risk of vitreous hemorrhage post-vitreoretinal surgery

The meta-analysis of vitreous hemorrhage included six unique studies [15–20], contributing eight independent data comparisons (Fabinyi et al. and Wang et al. each contributed data to both the aspirin versus control and the continuation versus discontinuation subgroups), with a total of 548 patients in the experimental/aspirin groups and 2,019 in the control/comparator groups across both subgroups. A subgroup analysis was performed comparing aspirin versus control (no aspirin) and aspirin continuation versus discontinuation (Fig. 2).

For the aspirin versus control subgroup, which included six studies [15–20], the pooled risk ratio was 1.58 (95% CI: 0.66, 3.82), indicating no statistically significant increase in vitreous hemorrhage risk with aspirin use. The analysis revealed moderate heterogeneity within this subgroup (I² = 67.9%, tau² = 0.79, Q = 15.57), suggesting some variability in effect estimates across the included studies. Despite this heterogeneity, the confidence interval clearly encompasses 1.0, demonstrating that aspirin use compared to control does not significantly increase the risk of vitreous hemorrhage following vitreoretinal surgery.

The continuation versus discontinuation subgroup comprised two studies [18, 20] and yielded a pooled risk ratio of 1.06 (95% CI: 0.08, 13.34), similarly showing no significant difference between continuation and discontinuation strategies. This subgroup also demonstrated moderate heterogeneity (I² = 62.4%, tau² = 2.25, Q = 2.66). The wide confidence interval reflects the limited number of studies and small event rates in this comparison, indicating substantial uncertainty in the point estimate. However, the risk ratio near 1.0 suggests no meaningful difference between aspirin continuation and discontinuation approaches.

Importantly, the test for subgroup differences showed no significant variation between the aspirin versus control and continuation versus discontinuation comparisons (Q = 0.08, *p* = 0.771), indicating consistency in the lack of effect across different comparison types. The overall heterogeneity across all comparisons was moderate (I² = 61.7%, tau² = 0.68, Q = 18.27, *p* = 0.011).

To contextualize the clinical relevance of these findings, the absolute risk difference for vitreous hemorrhage was calculated using the pooled control group event rate as baseline. The baseline rate of vitreous hemorrhage in control groups was 3.71%, and the pooled RR of 1.55 corresponds to an absolute risk difference of 1.76% (17.6 additional events per 1,000 surgeries; NNH = 56.8), which was not statistically significant.

### The risk of hyphema post-vitreoretinal surgery

**Fig. 3:**
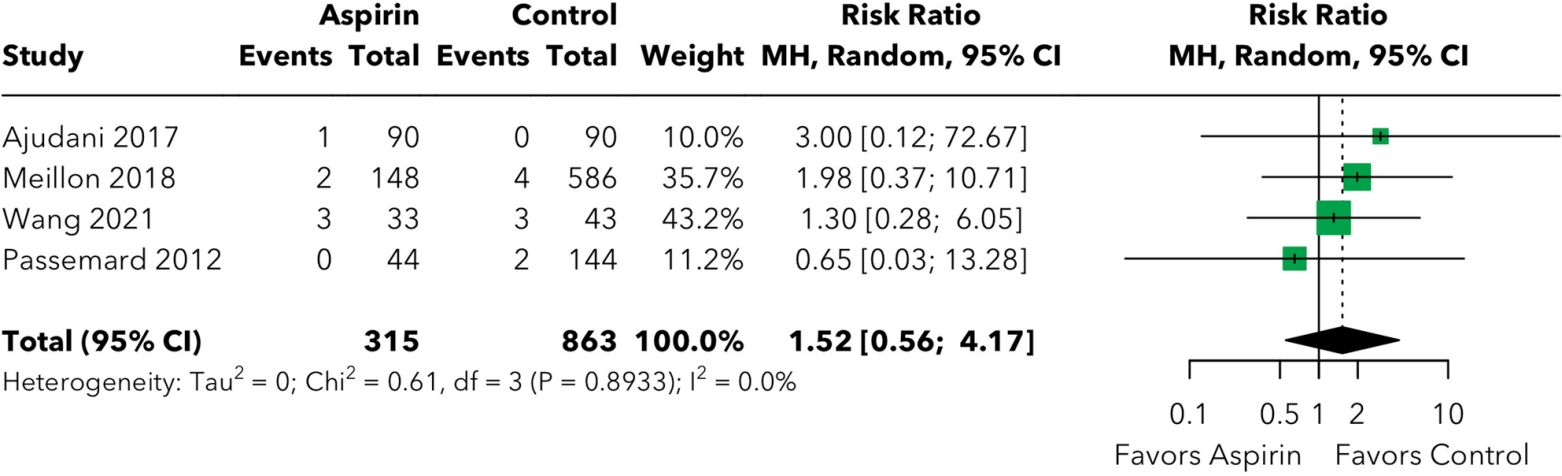
The risk of hyphema post-vitreoretinal surgery

The analysis of hyphema included four studies [15–18] with a total of 1,178 patients (315 in the experimental group and 863 in the control group), with 15 total events reported across all studies (Fig. 3). The pooled analysis demonstrated no significant increase in hyphema risk associated with aspirin continuation, with a risk ratio of 1.52 (95% CI: 0.56, 4.17; *p* = 0.415).

Individual study risk ratios ranged from 0.65 (95% CI: 0.03, 13.28) in Passemard 2012 [17] to 3.00 (95% CI: 0.12, 72.67) in Ajudani 2017 [15]. The analysis showed excellent homogeneity across studies, with no evidence of heterogeneity (I² = 0.0%, tau² = 0, Q = 0.61, *p* = 0.893). The absolute risk difference for hyphema was 0.86% (8.6 additional events per 1,000 surgeries; NNH = 116.0), representing a clinically modest and statistically nonsignificant increase.

### The risk of other hemorrhagic complications post-vitreoretinal surgery

**Fig. 4:**
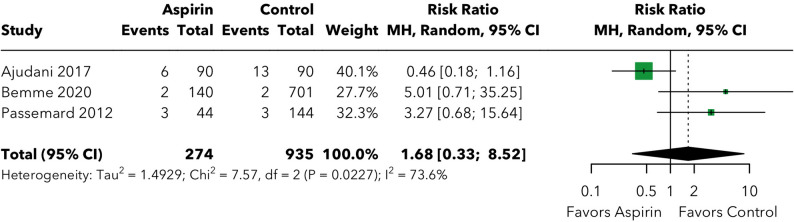
The risk of other hemorrhagic complications post-vitreoretinal surgery, stratified by complication type (subconjunctival hemorrhage, subretinal hemorrhage, choroidal hemorrhage, and retinal hemorrhage)

Subconjunctival, subretinal, choroidal, and retinal hemorrhage were analyzed within a single combined forest plot with subgroup analyses by hemorrhage type (Fig. 4). Results are reported for each subtype individually below.

For **subconjunctival hemorrhage**, two studies [15, 17] contributed data (368 patients; 134 in the aspirin group and 234 in the control group). The pooled risk ratio was 1.20 (95% CI: 0.58–2.48; *p* = 0.622), with no evidence of heterogeneity (I² = 0.0%, tau² = 0, Q = 0.03). The absolute risk difference was 4.04% (40.4 additional events per 1,000 surgeries; NNH = 24.8), which did not reach statistical significance. Sensitivity analysis was not performed given the small number of contributing studies (k = 2).

For **subretinal hemorrhage**, three studies [15, 16, 19] contributed data (1,755 patients; 378 in the aspirin group and 1,377 in the control group). The pooled risk ratio was 1.06 (95% CI: 0.15–7.63; *p* = 0.956), with substantial heterogeneity (I² = 82.4%, tau² = 2.58, Q = 11.36). The absolute risk difference was − 1.40% (− 14.0 events per 1,000 surgeries; NNH = − 71.4), suggesting a numerically non-significant trend toward slightly fewer subretinal hemorrhages with aspirin; this should not be interpreted as a protective effect given the extremely wide confidence interval and substantial heterogeneity.

For **choroidal hemorrhage**, three studies [7, 16, 19] contributed data (2,109 patients; 348 in the aspirin group and 1,761 in the control group). The pooled risk ratio was 2.07 (95% CI: 0.81–5.24; *p* = 0.127), with no evidence of heterogeneity (I² = 0.0%, tau² = 0, Q = 0.27). The absolute risk difference was 0.53% (5.3 additional events per 1,000 surgeries; NNH = 188.1). While the point estimate suggests a possible non-significant trend toward increased choroidal hemorrhage risk, this did not reach statistical significance. Sensitivity analysis was not performed given the absence of meaningful heterogeneity (I² = 0%).

For **retinal hemorrhage**, three studies [15, 17, 19] contributed data (1,209 patients; 274 in the aspirin group and 935 in the control group). The pooled risk ratio was 1.68 (95% CI: 0.33–8.52; *p* = 0.532), with substantial heterogeneity (I² = 73.6%, tau² = 1.49, Q = 7.57). The absolute risk difference was 2.09% (20.9 additional events per 1,000 surgeries; NNH = 47.9), which was not statistically significant.

The test for subgroup differences across the four hemorrhage subtypes showed no significant variation (Q = 0.944, *p* = 0.815), indicating that the null effect of aspirin was consistent regardless of the anatomical location of the hemorrhage.

### Visual acuity changes post-vitreoretinal surgery

**Fig. 5:**
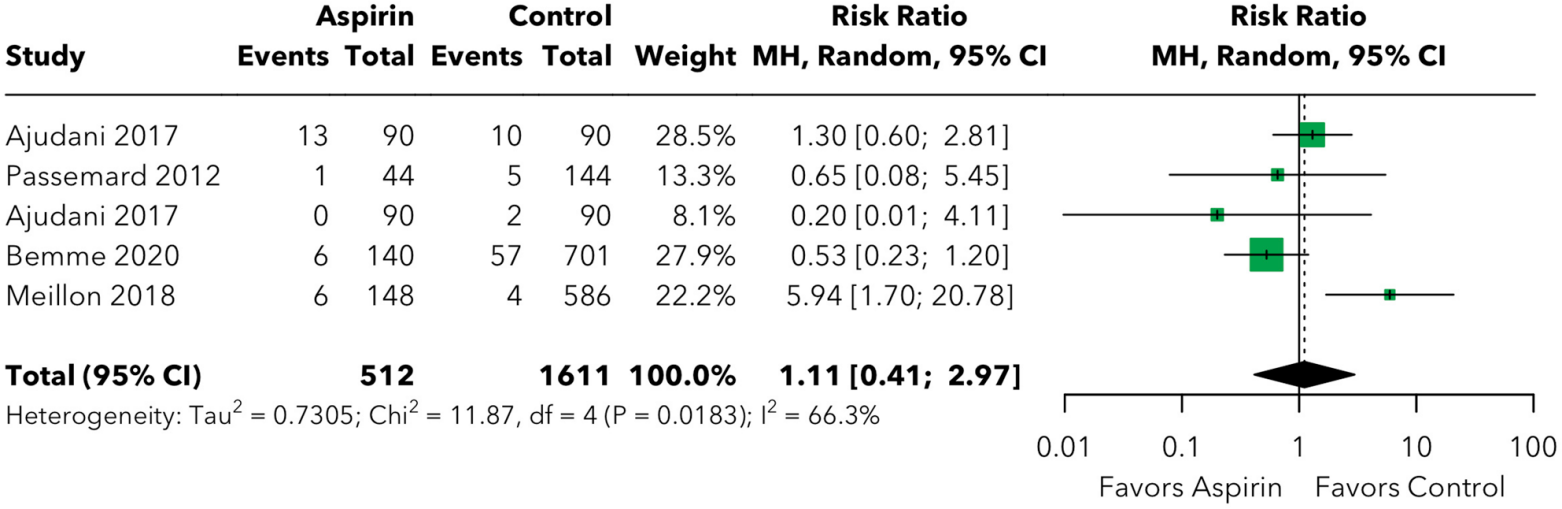
Visual acuity changes post-vitreoretinal surgery

Two studies [17, 18] with a total of 264 patients (77 in the experimental group and 187 in the control group) assessed postoperative visual acuity changes (Fig. 5). The pooled analysis demonstrated no statistically significant difference in postoperative visual acuity between patients who continued aspirin and those in the control groups (mean difference [MD] = 0.26 logMAR, 95% CI: −0.005 to 0.52; *p* = 0.055).

The individual studies showed mean differences of 0.12 (95% CI: -0.17, 0.41) in Wang 2020 [18] and 0.39 (95% CI: 0.12, 0.66) in Passemard 2012 [17]. The analysis revealed moderate heterogeneity (I² = 42.8%, tau² = 0.016, Q = 1.75, *p* = 0.186), suggesting some variability in effect estimates between studies. Notably, the upper bound of the 95% CI (0.52 logMAR) exceeds the established minimal clinically important difference (MCID) of 0.2 logMAR, indicating that clinical significance cannot be entirely excluded despite the absence of statistical significance. Table 2 summarizes the main findings of our meta-analysis.

**Table 2:** Summary of meta-analysis results

Outcome	Studies (*n*)	Patients (*n*)	RR/MD	95% CI	*P*-value	I² (%)	Interpretation
Vitreous Hemorrhage	7	1,745	1.55	0.73–3.29	0.253	61.7	No significant difference
Hyphema	4	1,178	1.52	0.56–4.17	0.415	0.0	No significant difference
Retrobulbar Hemorrhage	3	1,209	1.68	0.33–8.52	0.532	73.6	No significant difference
Subconjunctival Hemorrhage	5	2,123	1.11	0.41–2.97	0.835	66.3	No significant difference
Visual Acuity	2	264	0.26*	-0.01–0.52	0.055	42.8	No significant difference

*Mean Difference (MD) reported for Visual Acuity; Risk Ratio (RR) reported for all other outcomes. CI = Confidence Interval

### Quality assessment, sensitivity analyses, and publication bias

Quality assessment using the Newcastle-Ottawa Scale (NOS) revealed that four studies (57.1%) were rated as high quality (7–9 stars): Ajudani 2017 [15], Meillon 2018 [16], Bemme 2020 [19], and Wang 2020 [18] (Supplementary Table S2). Three studies (42.9%) were rated as moderate quality (4–6 stars): Narendran 2003 [7], Fabinyi 2011 [20], and Passemard 2012 [17]. No studies were classified as low quality. The moderate-quality studies primarily lost points due to lack of representativeness of the exposed cohort, inadequate control for confounding factors, or insufficient follow-up duration. The predominance of high-quality studies strengthens confidence in the meta-analysis findings, though the observational nature of all included studies limits the ability to establish definitive causal relationships.

Leave-one-out sensitivity analyses were performed for outcomes with substantial heterogeneity (I² > 50%): retinal hemorrhage (I² = 73.6%), subretinal hemorrhage (I² = 82.4%), and the vitreous hemorrhage aspirin versus control subgroup (I² = 67.9%) (Supplementary Table S3). For retinal hemorrhage, pooled risk ratios ranged from 1.11 to 3.87 across iterations, with heterogeneity driven by opposing individual study effects in Ajudani 2017 [15] (RR = 0.46) and Bemme 2020 [19] (RR = 5.01). For subretinal hemorrhage, sensitivity analyses demonstrated instability attributable to the small number of contributing studies and low event rates, with no single study dominating the pooled estimate. For vitreous hemorrhage, risk ratios ranged from 1.22 to 2.21 across leave-one-out iterations, with five of six iterations remaining non-significant. All sensitivity analyses maintained confidence intervals encompassing or approaching 1.0, supporting the robustness of findings.

Publication bias was assessed using Doi plots and the Luis Furuya-Kanamori (LFK) index for all outcomes (Supplementary Table S4). No asymmetry was detected for hyphema (LFK = 0.85), the combined other hemorrhagic complications outcome overall (LFK = − 0.35), subretinal hemorrhage (LFK = 0.08), vitreous hemorrhage overall (LFK = − 0.76), and the vitreous hemorrhage aspirin versus control subgroup (LFK = 0.25). Major asymmetry was observed for retinal hemorrhage (LFK = 4.32), choroidal hemorrhage (LFK = − 2.73), and subconjunctival hemorrhage (LFK = − 3.93); however, these findings lack sufficient validity given the small number of studies contributing to each subtype (k ≤ 3), as LFK index calculations require larger sample sizes for reliable asymmetry detection. The vitreous hemorrhage continuation versus discontinuation subgroup showed major asymmetry (LFK = − 3.74), similarly limited by only two contributing studies (k = 2). Visual acuity demonstrated minor asymmetry (LFK = − 1.82). The absence of asymmetry in the primary outcome and the most well-studied outcomes suggests minimal impact of publication bias on the primary conclusions.

### Certainty of evidence (GRADE assessment)

The certainty of evidence for each outcome was formally assessed using the GRADE framework (Supplementary Table S5). Due to the predominantly observational design of included studies (five cohort studies and one retrospective case series, alongside one randomized controlled trial), the baseline certainty for most outcomes was rated as low. Downgrading was applied for inconsistency when I² exceeded 50% and for imprecision when confidence intervals were wide, crossed the null, or were based on few contributing studies.

For the primary outcome, vitreous hemorrhage, the certainty of evidence was rated as **Very Low**, owing to moderate inconsistency (I² = 61.7%) and imprecision of the pooled estimate (95% CI: 0.73–3.29). Hyphema and choroidal hemorrhage were rated as **Low** certainty, given acceptable homogeneity but wide confidence intervals. Subretinal hemorrhage (I² = 82.4%), retinal hemorrhage (I² = 73.6%), subconjunctival hemorrhage (k = 2 studies only), and visual acuity (k = 2 studies only) were each rated as **Very Low** certainty, due to very high heterogeneity, very wide confidence intervals, or insufficient contributing studies.

These certainty ratings underscore that the finding of no statistically significant difference in hemorrhagic complications with aspirin continuation must be interpreted with caution. The current body of evidence is best characterized as hypothesis-generating, and high-quality prospective randomized data are needed to confirm or refute this conclusion.

## Discussion

This systematic review and meta-analysis evaluated the safety of aspirin continuation versus discontinuation in patients undergoing vitreoretinal surgery. Our principal finding is that aspirin continuation was not associated with a statistically significant increase in hemorrhagic complications compared to aspirin discontinuation or no aspirin use. Specifically, we found no significant differences in rates of vitreous hemorrhage, hyphema, subconjunctival hemorrhage, subretinal hemorrhage, choroidal hemorrhage, or retinal hemorrhage. However, given the predominantly observational nature of included evidence and the low to very low certainty of evidence across all outcomes per GRADE assessment, these findings should be considered hypothesis-generating and interpreted with appropriate caution, pending confirmation from high-quality prospective randomized data.

Our findings in vitreoretinal surgery align with the recent comprehensive meta-analysis by Abo Zeid et al., examining aspirin use in cataract surgery [11]. In their analysis of 65,196 patients, they found that while aspirin continuation increased the risk of subconjunctival hemorrhage, it did not increase the risk of sight-threatening complications such as hyphema, retrobulbar hemorrhage, or vitreous hemorrhage. Importantly, they recommended continuation of aspirin in patients undergoing cataract surgery. Our study extends these findings to the more complex realm of vitreoretinal surgery, where concerns about bleeding complications might intuitively seem greater due to the more invasive nature of the procedures and the presence of pathological neovascularization in many cases. It must be recognized, however, that the evidence base for vitreoretinal surgery is considerably smaller and of lower certainty than that for cataract surgery, and extrapolation of conclusions should therefore be made with appropriate caution.

Our meta-analysis builds upon and synthesizes findings from several individual studies examining aspirin use in vitreoretinal surgery. Narendran and Williamson [7] prospectively studied 541 patients undergoing vitreoretinal surgery and found that aspirin had little effect on bleeding during surgery, though they noted that warfarin was associated with bleeding complications. Our pooled analysis supports their conclusion regarding aspirin safety. Several subsequent studies have reported conflicting results. Passemard et al. [17] examined 206 patients undergoing vitreoretinal surgery and found that while overall hemorrhagic complications were similar between groups, potential sight-threatening complications were more frequent in patients receiving antiplatelet agents. In contrast, Oh et al. [8] studied 822 patients and concluded that antiplatelet therapy could be continued safely during vitreoretinal surgery, though they noted a trend toward increased complications that did not reach statistical significance.

More recent studies have further supported aspirin continuation. Wang et al. [18] conducted a prospective randomized trial of 110 patients and found no significant difference in hemorrhagic complications between aspirin continuation and discontinuation groups. Meillon et al. [16]. in a large multicenter study of 804 patients found that while antiplatelet agents showed increased complications in univariate analysis, this association disappeared in multivariate analysis, with only endodiathermy use remaining significant — highlighting that intraoperative hemostatic technique may be a more important determinant of bleeding than antiplatelet status itself [16]. Similarly, Ajudani et al. [15] found no significant difference in bleeding complications between the aspirin and control groups. Bemme et al. [19] focused specifically on rhegmatogenous retinal detachment surgery and found no significantly increased rate of perioperative hemorrhages under aspirin compared with controls, though they noted that scleral buckling with subretinal fluid drainage showed the highest hemorrhage rates regardless of anticoagulation status. This highlights an important point: surgical technique and specific procedural factors may be more important determinants of bleeding risk than antiplatelet therapy itself.

The management of aspirin therapy before vitreoretinal surgery represents a clinical dilemma where the risks of continuation must be weighed against the risks of discontinuation. The thrombotic risks associated with aspirin discontinuation are well-established and potentially catastrophic. Gerstein et al. [5] demonstrated that thromboembolic complications following aspirin cessation can occur with median onset times as brief as 8.5 days, with events including myocardial infarction, stroke, and stent thrombosis. The concept of aspirin rebound has been described [6], characterized by increased platelet reactivity creating a prothrombotic state following withdrawal. The implications of these thrombotic events extend far beyond the ophthalmic realm. A perioperative myocardial infarction or stroke carries significant mortality risk and may result in permanent disability that far outweighs any visual morbidity from surgical bleeding complications. Furthermore, the delay in surgery necessitated by aspirin discontinuation protocols may itself compromise visual outcomes, particularly in time-sensitive conditions such as macula-involving retinal detachments [10]. 

Vitreoretinal surgery might theoretically carry higher bleeding risks than anterior segment procedures due to several factors, including the creation of sclerotomies, the manipulation of pathological tissues, periods of controlled hypotony, and the presence of abnormal neovascularization in many cases. However, our meta-analysis suggests that these theoretical concerns do not translate into clinically significant increased bleeding complications with aspirin continuation. This may be explained by several factors. Modern vitreoretinal surgical techniques have evolved to minimize trauma, with small-gauge instrumentation becoming standard. Improved hemostatic techniques, including endodiathermy, provide effective intraoperative bleeding control during vitreoretinal procedures [16]. The bleeding complications that do occur are often minor and self-limited, rarely affecting final visual outcomes.

The generalizability of our findings warrants careful consideration. The majority of included studies originated from high-income countries with access to modern small-gauge instrumentation, standardized anesthetic protocols, and experienced vitreoretinal surgeons. Surgical technique was a more significant predictor of hemorrhagic risk than antiplatelet status in several included studies — most notably in Bemme et al., where scleral buckling with subretinal fluid drainage carried significantly higher bleeding rates regardless of anticoagulation status. In resource-limited settings where 20-gauge systems remain standard, where complex fibrovascular dissection is performed more frequently without the availability of adjunct anti-VEGF therapy, or where access to advanced hemostatic equipment and surgeon experience may differ, the risk profile may not be identical. Surgeons practicing in such settings should exercise individualized clinical judgment, particularly for higher-risk procedures such as proliferative diabetic retinopathy vitrectomy with extensive delamination, rather than applying a uniform continuation policy. Similarly, patients with active neovascularization, prior vitreoretinal surgery, or need for extensive membrane dissection represent subgroups in whom individualized risk-benefit assessment is most warranted.

Based on our findings, which are predominantly derived from observational studies and thus carry low to very low certainty of evidence per GRADE, we suggest that aspirin therapy can likely be continued in most patients undergoing vitreoretinal surgery without a significantly increased risk of hemorrhagic complications. This recommendation should be considered hypothesis-generating and interpreted cautiously, pending confirmation by high-quality prospective randomized data. It is also worth noting that in current clinical practice, many patients on aspirin therapy are routinely asked to discontinue it three or more days before vitreoretinal surgery — a practice that our findings suggest may expose patients to avoidable thromboembolic risk without a clearly demonstrated reduction in surgical bleeding.

This meta-analysis has several important limitations. The observational nature of most included studies (only one randomized controlled trial [18]) limits our ability to establish definitive causal relationships and introduces potential selection bias. Substantial heterogeneity existed across studies in surgical indications, techniques (varying gauge instrumentation and use of scleral buckling), anesthetic approaches, and outcome definitions, which contributed to moderate statistical heterogeneity in several outcomes. The comparison strategies varied, with some studies comparing aspirin use versus no antiplatelet therapy and others comparing continuation versus discontinuation, potentially capturing different aspects of risk. The relatively small number of studies for each outcome (2–7 studies) limits statistical power and the reliability of publication bias assessment. Limited data were available on aspirin dosage, timing protocols, and management of other antiplatelet agents, particularly clopidogrel and dual antiplatelet therapy. Most studies were conducted in developed countries, potentially limiting generalizability to resource-limited settings. The included studies did not consistently differentiate intraoperative from postoperative hemorrhagic events, nor uniformly distinguish minor self-limiting hemorrhages from those requiring unplanned surgical reintervention, precluding formal subgroup analysis by hemorrhage timing or clinical severity. Furthermore, the definitions of vitreous hemorrhage and other hemorrhagic outcomes were not standardized across studies — with some defining hemorrhage by clinical appearance and others by the requirement for surgical reintervention — contributing to the moderate heterogeneity observed. A formal GRADE assessment confirms overall certainty of evidence as low to very low across all outcomes, and the recommendation for aspirin continuation should be interpreted accordingly. Additionally, gray literature sources, including ClinicalTrials.gov and major conference proceedings, were not searched; however, the four peer-reviewed databases used provide comprehensive coverage of the published clinical literature, and the absence of significant publication bias in primary outcomes (LFK index for overall VH: −0.76) provides some reassurance.

Future research should prioritize prospective randomized trials stratified by cardiovascular risk category (high versus low risk for thromboembolic events), surgical complexity (routine macular surgery versus proliferative diabetic retinopathy with extensive fibrovascular dissection), and aspirin indication (primary versus secondary prevention). Standardized outcome definitions — particularly for vitreous hemorrhage severity and the threshold for surgical reintervention — are urgently needed to enable meaningful cross-study comparison. Patient-level registries capturing detailed cardiovascular risk profiles, aspirin dosage and timing protocols, and both hemorrhagic and systemic thrombotic outcomes would provide the granular data necessary to develop evidence-based, risk-stratified perioperative guidelines. Studies should also specifically address higher-risk patient subgroups and surgical scenarios, including those common in resource-limited settings where modern small-gauge technology may not be universally available.

## Conclusion

This study demonstrates that aspirin continuation does not significantly increase the risk of hemorrhagic complications in patients undergoing vitreoretinal surgery. No significant differences were observed in rates of vitreous hemorrhage, hyphema, subconjunctival hemorrhage, subretinal hemorrhage, choroidal hemorrhage, retinal hemorrhage, or postoperative visual acuity between patients continuing aspirin therapy and those discontinuing it or not receiving antiplatelet therapy. While these findings support a general recommendation for aspirin continuation in most patients undergoing vitreoretinal surgery, the predominantly observational nature of the evidence and the low to very low-GRADE certainty across all outcomes limit definitive causal inference. These findings are best interpreted as hypothesis-generating and supportive of a clinical shift toward aspirin continuation, pending confirmation by high-quality prospective randomized data.

## Supplementary Information

Below is the link to the electronic supplementary material.

Supplementary Material 1

## Data Availability

No datasets were generated or analysed during the current study.
