# Supplementary material for: Do we need to hold aspirin before vitreoretinal surgery? A systematic review and meta-analysis
Source: Int J Retina Vitreous. 2026 Mar 31;12:74. doi: 10.1186/s40942-026-00840-3 (PMC13162363; doi:10.1186/s40942-026-00840-3)
Supplement: Supplementary file 1 — Supplementary Material 1 [file 40942_2026_840_MOESM1_ESM.docx]

# **Supplementary Material**

Do We Need to Hold Aspirin Before Vitreoretinal Surgery? A Systematic Review and Meta-Analysis

## **Supplementary Table S1:** Detailed Database Search Strategy and Results

| **Database** | **Search String** | **Results (n)** | **Percentage (%)** |
| --- | --- | --- | --- |
| **PubMed/MEDLINE** | ((("Vitrectomy"[Mesh]) OR ("Retinal Detachment"[Mesh:NoExp]/surgery) OR ("Epiretinal Membrane"[Mesh]/surgery) OR ("Retinal Perforations"[Mesh]/surgery) OR ("Diabetic Retinopathy"[Mesh]/surgery) OR ("vitrectomy"[tiab]) OR ("vitreoretinal surgery"[tiab]) OR ("vitreoretinal surgeries"[tiab]) OR ("pars plana vitrectomy"[tiab]) OR ("PPV"[tiab]) OR ("retinal surgery"[tiab]) OR ("retinal detachment surgery"[tiab]) OR ("retinal detachment repair"[tiab]) OR ("macular hole surgery"[tiab]) OR ("epiretinal membrane surgery"[tiab]) OR ("diabetic vitrectomy"[tiab]) OR ("scleral buckling"[tiab]) OR ("retinal reattachment"[tiab]) OR ("vitreoretinal procedure*"[tiab]) OR ("posterior segment surgery"[tiab])) AND (("Aspirin"[Mesh]) OR ("Platelet Aggregation Inhibitors"[Mesh]) OR ("aspirin"[tiab]) OR ("acetylsalicylic acid"[tiab]) OR ("ASA"[tiab]) OR ("antiplatelet"[tiab]) OR ("anti-platelet"[tiab]) OR ("antiplatelet agent*"[tiab]) OR ("antiplatelet therap*"[tiab]) OR ("platelet inhibitor*"[tiab]) OR ("platelet aggregation inhibitor*"[tiab]) OR ("antithrombotic*"[tiab]) OR ("clopidogrel"[tiab]) OR ("ticagrelor"[tiab]) OR ("prasugrel"[tiab])) AND (("Perioperative Care"[Mesh]) OR ("Preoperative Care"[Mesh]) OR ("perioperative"[tiab]) OR ("preoperative"[tiab]) OR ("pre-operative"[tiab]) OR ("postoperative"[tiab]) OR ("post-operative"[tiab]) OR ("intraoperative"[tiab]) OR ("continuation"[tiab]) OR ("discontinuation"[tiab]) OR ("withdrawal"[tiab]) OR ("cessation"[tiab]) OR ("withholding"[tiab]) OR ("management"[tiab]) OR ("hemorrhag*"[tiab]) OR ("haemorrhag*"[tiab]) OR ("bleeding"[tiab]) OR ("complication*"[tiab]) OR ("safety"[tiab]) OR ("adverse event*"[tiab]))) NOT (("Animals"[Mesh] NOT "Humans"[Mesh]) OR ("Case Reports"[pt]) OR ("comment"[pt]) OR ("editorial"[pt]) OR ("news"[pt]) OR ("letter"[pt])) AND English[lang] | 145 | 16.6 |
| **Ovid (MEDLINE, Embase)** | ((exp Vitrectomy/ or exp Retinal Detachment/su or exp Epiretinal Membrane/su or exp Retinal Perforations/su or exp Diabetic Retinopathy/su or vitrectomy.mp. or vitreoretinal surgery.mp. or vitreoretinal surgeries.mp. or pars plana vitrectomy.mp. or PPV.mp. or retinal surgery.mp. or retinal detachment surgery.mp. or retinal detachment repair.mp. or macular hole surgery.mp. or epiretinal membrane surgery.mp. or diabetic vitrectomy.mp. or scleral buckling.mp. or retinal reattachment.mp. or vitreoretinal procedure$.mp. or posterior segment surgery.mp.) and (exp Aspirin/ or exp Platelet Aggregation Inhibitors/ or aspirin.mp. or acetylsalicylic acid.mp. or ASA.mp. or antiplatelet.mp. or anti-platelet.mp. or antiplatelet agent$.mp. or antiplatelet therap$.mp. or platelet inhibitor$.mp. or platelet aggregation inhibitor$.mp. or antithrombotic$.mp. or clopidogrel.mp. or ticagrelor.mp. or prasugrel.mp.) and (exp Perioperative Care/ or exp Preoperative Care/ or perioperative.mp. or preoperative.mp. or pre-operative.mp. or postoperative.mp. or post-operative.mp. or intraoperative.mp. or continuation.mp. or discontinuation.mp. or withdrawal.mp. or cessation.mp. or withholding.mp. or management.mp. or hemorrhag$.mp. or haemorrhag$.mp. or bleeding.mp. or complication$.mp. or safety.mp. or adverse event$.mp. or hyphema.mp. or vitreous hemorrhage.mp. or retrobulbar hemorrhage.mp. or subconjunctival hemorrhage.mp.)) not (exp animals/ not exp humans/ or case reports.pt. or comment.pt. or editorial.pt. or news.pt. or letter.pt.) and english.lg. | 155 | 17.8 |
| **Scopus** | (TITLE-ABS-KEY("vitrectomy" OR "vitreoretinal surgery" OR "vitreoretinal surgeries" OR "pars plana vitrectomy" OR "PPV" OR "retinal surgery" OR "retinal surgeries" OR "retinal detachment surgery" OR "retinal detachment repair" OR "macular hole surgery" OR "epiretinal membrane surgery" OR "diabetic vitrectomy" OR "scleral buckling" OR "retinal reattachment" OR "vitreoretinal procedure" OR "vitreoretinal procedures" OR "posterior segment surgery" OR "posterior segment surgeries" OR "retinal detachment repair" OR "pneumatic retinopexy" OR "vitreous surgery") AND TITLE-ABS-KEY("aspirin" OR "acetylsalicylic acid" OR "ASA" OR "antiplatelet" OR "anti-platelet" OR "antiplatelet agent" OR "antiplatelet agents" OR "antiplatelet therapy" OR "antiplatelet therapies" OR "platelet inhibitor" OR "platelet inhibitors" OR "platelet aggregation inhibitor" OR "platelet aggregation inhibitors" OR "antithrombotic" OR "antithrombotics" OR "clopidogrel" OR "ticagrelor" OR "prasugrel" OR "platelet aggregation inhibition") AND TITLE-ABS-KEY("perioperative" OR "preoperative" OR "pre-operative" OR "postoperative" OR "post-operative" OR "intraoperative" OR "continuation" OR "discontinuation" OR "withdrawal" OR "cessation" OR "withholding" OR "management" OR "hemorrhage" OR "hemorrhages" OR "haemorrhage" OR "haemorrhages" OR "bleeding" OR "complication" OR "complications" OR "safety" OR "adverse event" OR "adverse events" OR "adverse outcome" OR "adverse outcomes" OR "hyphema" OR "vitreous hemorrhage" OR "retrobulbar hemorrhage" OR "subconjunctival hemorrhage" OR "conjunctival hemorrhage")) AND NOT (DOCTYPE(re) OR DOCTYPE(ed) OR DOCTYPE(no) OR DOCTYPE(le) OR DOCTYPE(cr)) AND (LIMIT-TO(LANGUAGE,"English")) | 301 | 34.5 |
| **Web of Science** | ((TS=("vitrectomy") OR TS=("vitreoretinal surgery") OR TS=("vitreoretinal surgeries") OR TS=("pars plana vitrectomy") OR TS=("PPV") OR TS=("retinal surgery") OR TS=("retinal surgeries") OR TS=("retinal detachment surgery") OR TS=("retinal detachment repair") OR TS=("macular hole surgery") OR TS=("epiretinal membrane surgery") OR TS=("diabetic vitrectomy") OR TS=("scleral buckling") OR TS=("retinal reattachment") OR TS=("vitreoretinal procedure*") OR TS=("posterior segment surgery") OR TS=("posterior segment surgeries") OR TS=("pneumatic retinopexy") OR TS=("vitreous surgery")) AND (TS=("aspirin") OR TS=("acetylsalicylic acid") OR TS=("ASA") OR TS=("antiplatelet") OR TS=("anti-platelet") OR TS=("antiplatelet agent*") OR TS=("antiplatelet therap*") OR TS=("platelet inhibitor*") OR TS=("platelet aggregation inhibitor*") OR TS=("antithrombotic*") OR TS=("clopidogrel") OR TS=("ticagrelor") OR TS=("prasugrel")) AND (TS=("perioperative") OR TS=("preoperative") OR TS=("pre-operative") OR TS=("postoperative") OR TS=("post-operative") OR TS=("intraoperative") OR TS=("continuation") OR TS=("discontinuation") OR TS=("withdrawal") OR TS=("cessation") OR TS=("withholding") OR TS=("management") OR TS=("hemorrhag*") OR TS=("haemorrhag*") OR TS=("bleeding") OR TS=("complication*") OR TS=("safety") OR TS=("adverse event*") OR TS=("hyphema") OR TS=("vitreous hemorrhage") OR TS=("retrobulbar hemorrhage") OR TS=("subconjunctival hemorrhage"))) NOT (DT=(Review) NOT (TS=("systematic review") OR TS=("meta analysis") OR TS=("meta-analysis"))) AND LA=(English) | 271 | 31.1 |
| **Total (before deduplication)** | — | **872** | **100.0** |

**Filters Applied:**

- Language: English only
- Population: Human studies only (where applicable)
- Exclusions: Case reports, editorials, comments, letters, and news articles
- Animal studies excluded unless human data also present

## **Supplementary Table S2:** Quality Assessment of Included Studies Using the Newcastle-Ottawa Scale (NOS)

| **Study** | Representativeness of Exposed Cohort | Selection of Non-Exposed Cohort | Ascertainment of Exposure | Outcome Not Present at Start | Comparability of Cohorts* | Assessment of Outcome | Follow-up Duration Adequate | Adequacy of Follow-up | Total Score | Quality Rating |
| --- | --- | --- | --- | --- | --- | --- | --- | --- | --- | --- |
| Narendran 2003 | 1 | 1 | 1 | 1 | 1 | 1 | 0 | 0 | 6 | Moderate |
| Fabinyi 2011 | 0 | 1 | 1 | 1 | 0 | 1 | 1 | 1 | 6 | Moderate |
| Passemard 2012 | 0 | 1 | 1 | 1 | 0 | 1 | 1 | 1 | 6 | Moderate |
| Ajudani 2017 | 0 | 1 | 1 | 1 | 1 | 1 | 1 | 1 | 7 | High |
| Meillon 2018 | 1 | 1 | 1 | 1 | 2 | 1 | 1 | 1 | 9 | High |
| Bemme 2020 | 1 | 1 | 1 | 1 | 1 | 1 | 0 | 1 | 7 | High |
| Wang 2020 | 1 | 1 | 1 | 1 | 2 | 1 | 1 | 1 | 9 | High |

**Scoring System:**

- Each domain scored 0-1 point, except Comparability which can receive 0-2 points
- *Comparability assessed based on control for important confounders and additional factors

**Quality Classification Criteria:**

- **High Quality:** 7-9 stars
- **Moderate Quality:** 4-6 stars
- **Low Quality:** 0-3 stars

**Summary of Quality Assessment:**

- High Quality Studies: 4 (57.1%)
- Moderate Quality Studies: 3 (42.9%)
- Low Quality Studies: 0 (0%)

## **Supplementary Table S3:** Sensitivity Analysis (Leave-One-Out Analysis)

| **Figure Caption** | **Figure** |
| --- | --- |
| Sensitivity analysis for retrobulbar hemorrhage | 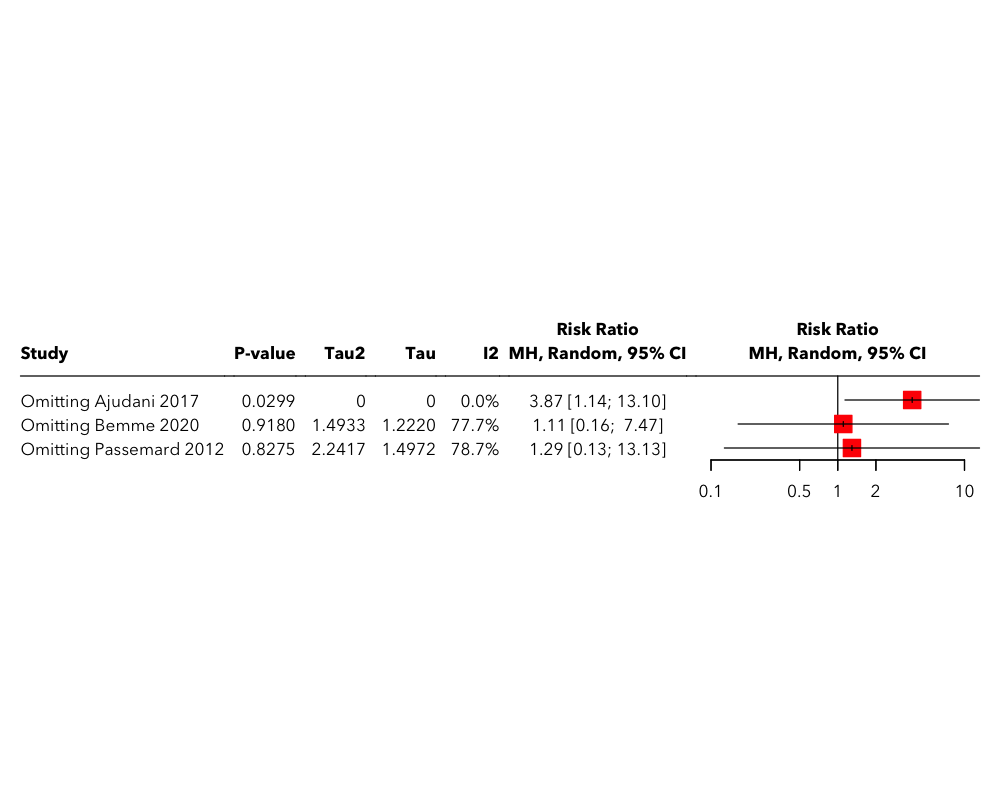 |
| Sensitivity analysis for subconjunctival hemorrhage | 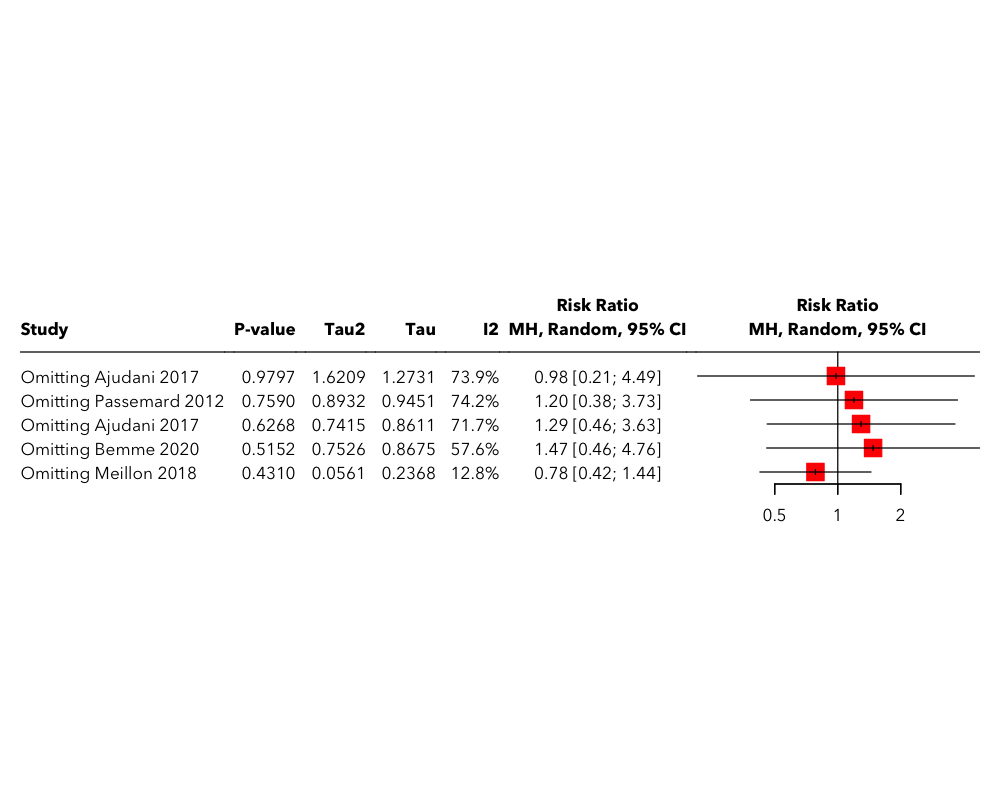 |
| Sensitivity analysis for vitreous hemorrhage (aspirin vs. control subgroup) | 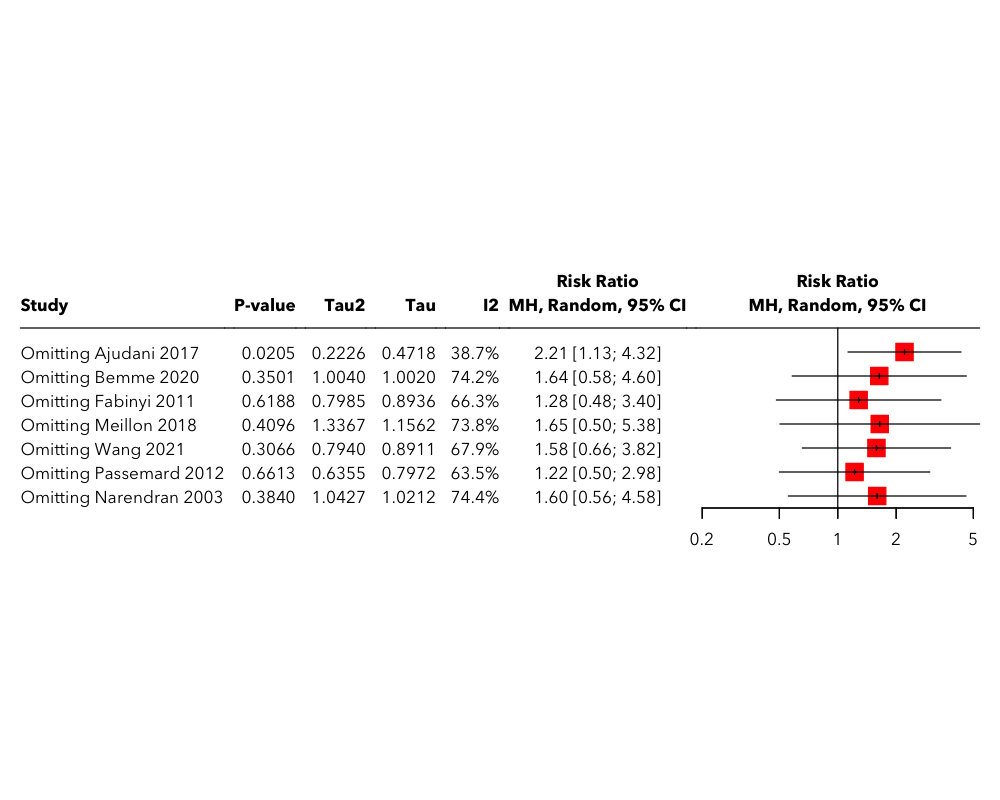 |

## **Supplementary Table S4:** Doi Plots for Assessment of Publication Bias

| **Figure Caption** | **Figure** |
| --- | --- |
|  |  |
| Doi plot for hyphema | 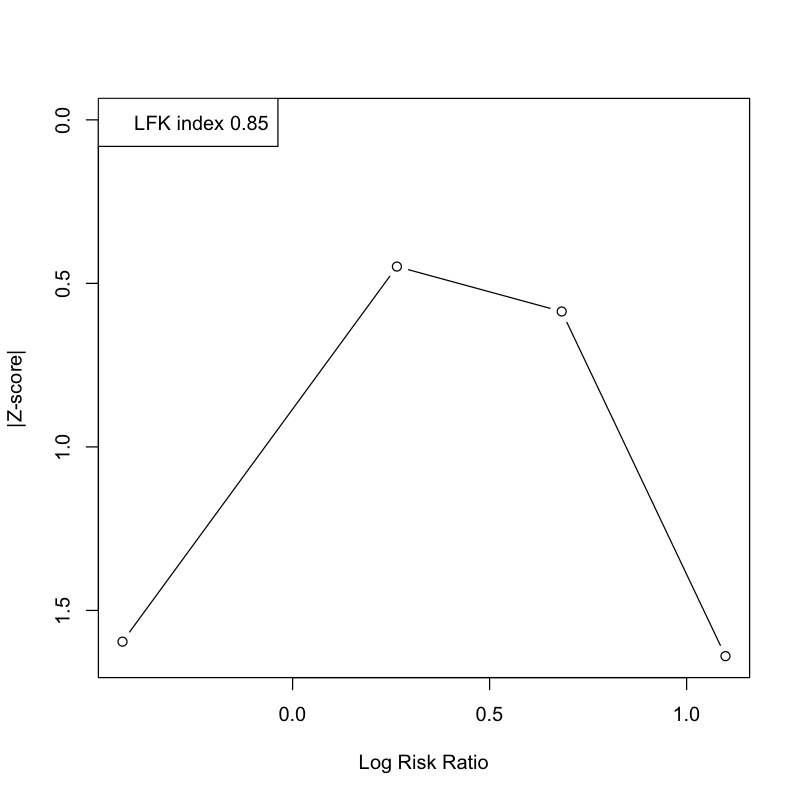 |
| Doi plot for retrobulbar hemorrhage | 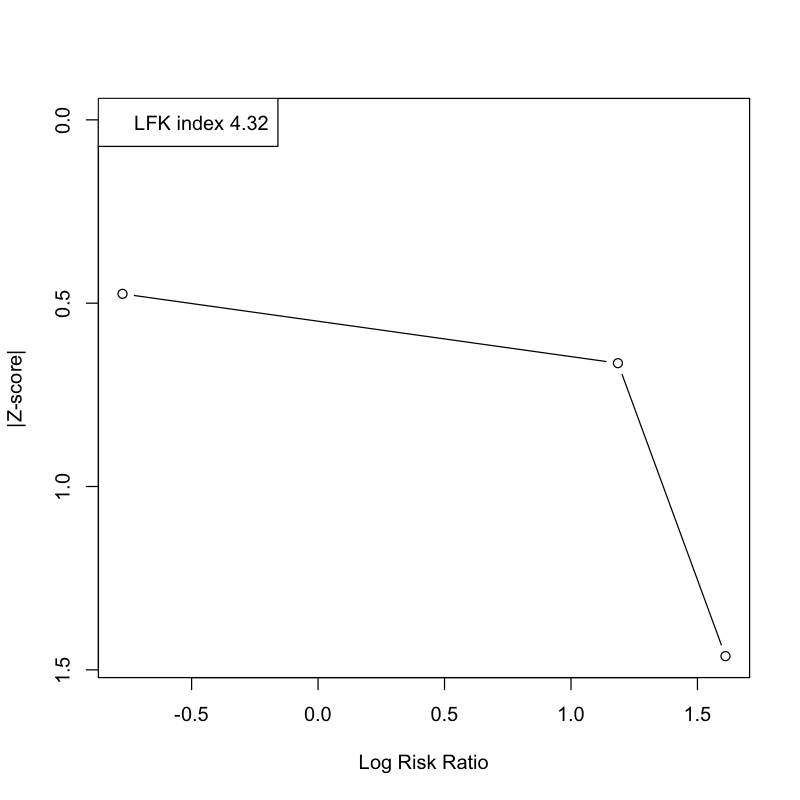 |
| Doi plot for subconjunctival hemorrhage | 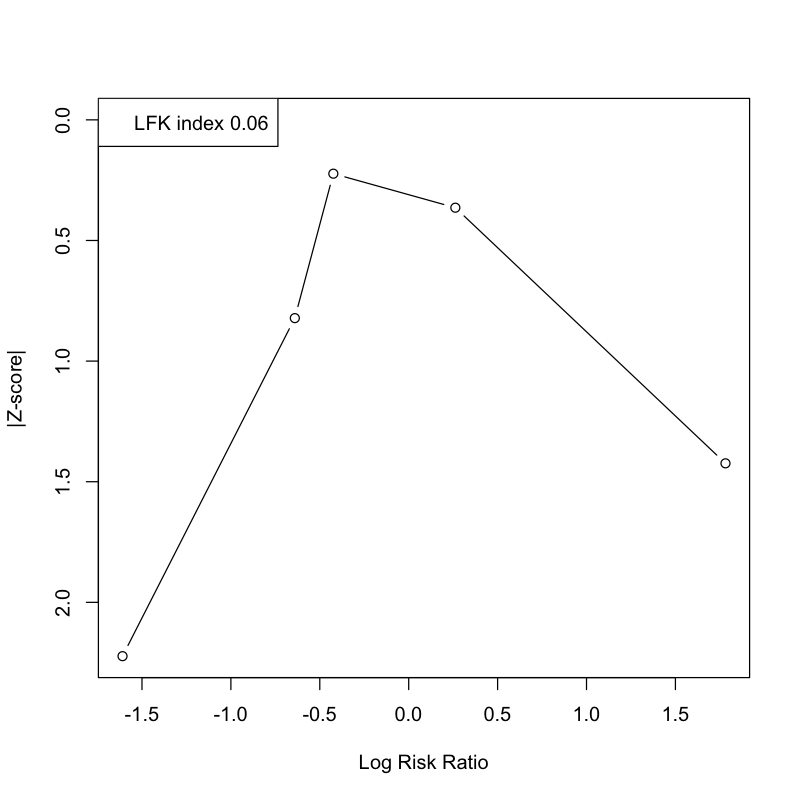 |
| Doi plot for vitreous hemorrhage (overall analysis) | 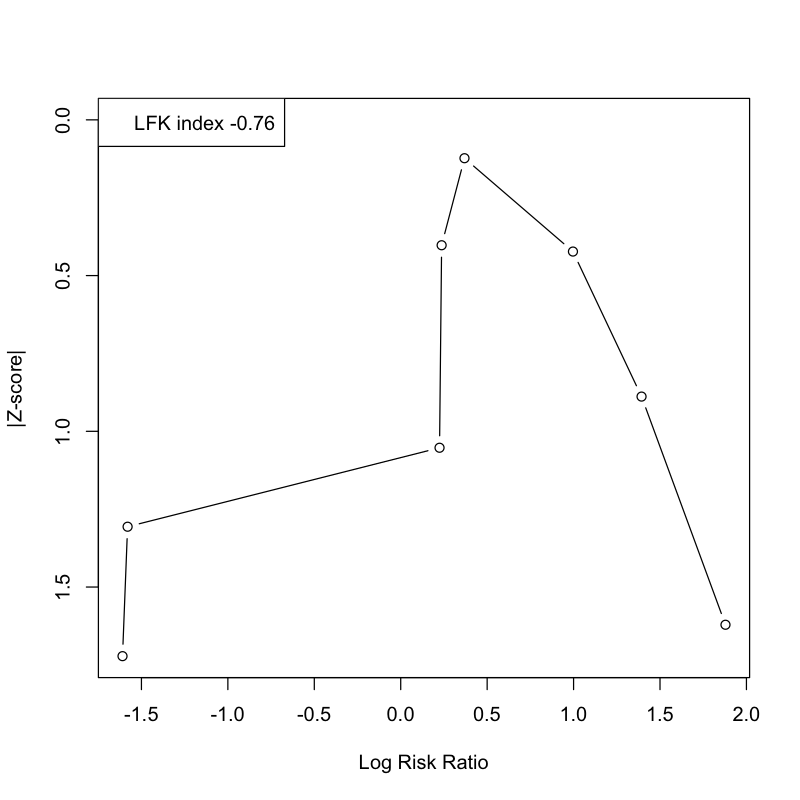 |
| Doi plot for vitreous hemorrhage (aspirin vs. control subgroup) | 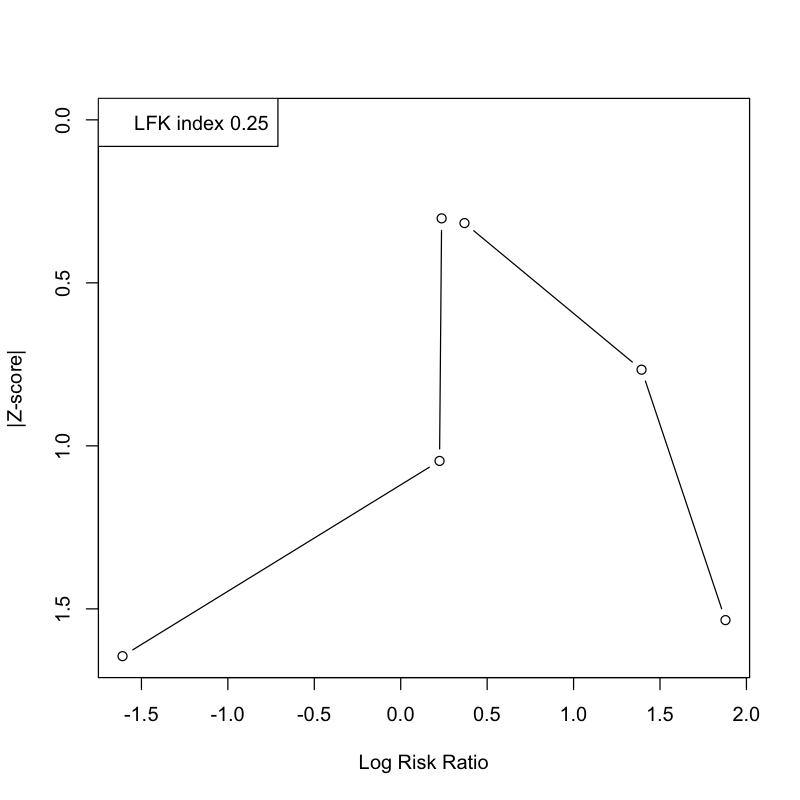 |
| Doi plot for vitreous hemorrhage (continuation vs. discontinuation subgroup) | 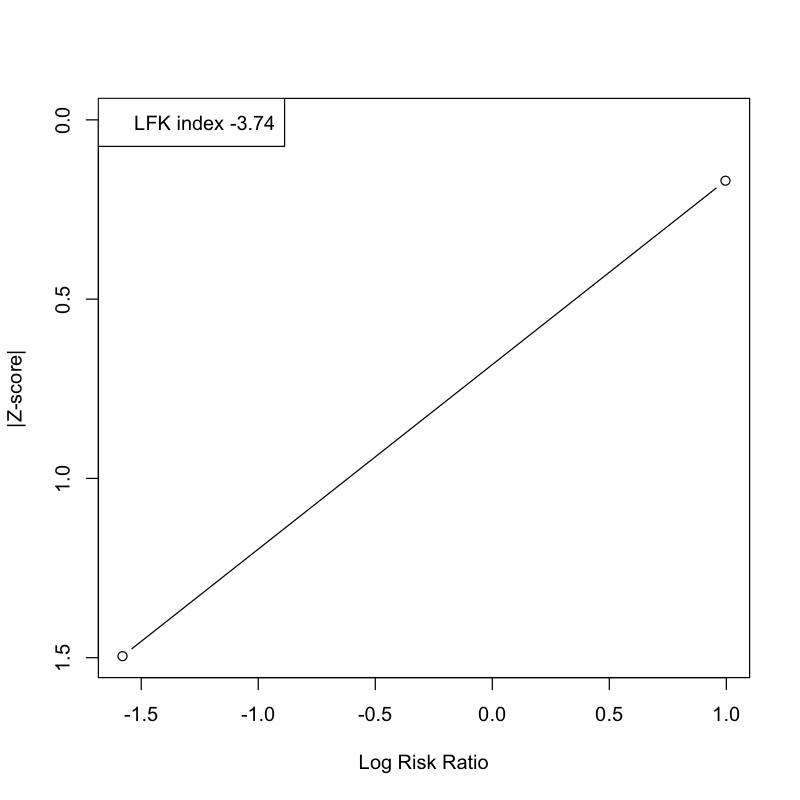 |
| Doi plot for visual acuity | 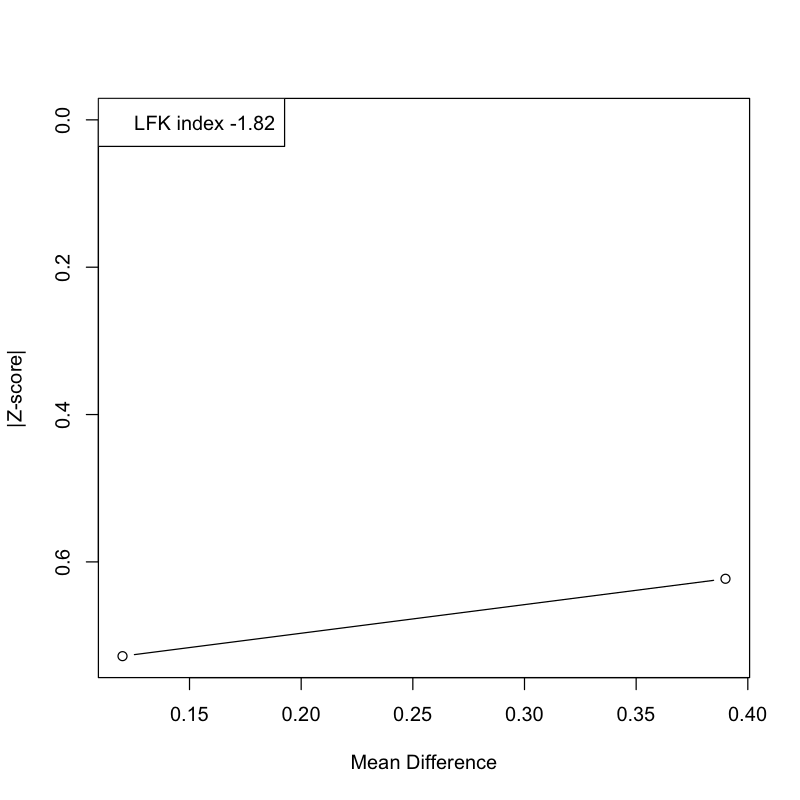 |

## Supplementary Notes

**Abbreviations:**

- ASA: Acetylsalicylic acid
- LFK: Luis Furuya-Kanamori (index)
- NOS: Newcastle-Ottawa Scale
- PPV: Pars plana vitrectomy
- RR: Risk ratio

**Statistical Methods:**

- Random-effects model used for all meta-analyses (DerSimonian-Laird method)
- Heterogeneity assessed using I² statistic and Cochran’s Q test
- Publication bias evaluated using Doi plots and LFK index
- Sensitivity analyses performed using leave-one-out method

**Interpretation of LFK Index:**

- LFK < ±1: No asymmetry (low risk of publication bias)
- LFK ±1 to ±2: Minor asymmetry
- LFK > ±2: Major asymmetry (high risk of publication bias)
